# The role of lncRNA-mediated ceRNA regulatory networks in pancreatic cancer

**DOI:** 10.1038/s41420-022-01061-x

**Published:** 2022-06-14

**Authors:** Jichuan Xu, Jian Xu, Xinyuan Liu, Jianxin Jiang

**Affiliations:** Department of Hepatobiliary Surgery, Renmin Hospital of Wuhan University, Wuhan, Hubei China

**Keywords:** Targeted therapies, Targeted therapies

## Abstract

Non-coding RNAs (ncRNAs), which occupy the vast majority of human transcripts are known for their inability to encode proteins. NcRNAs consist of a diverse range of RNA species, including long non-coding RNAs (lncRNAs), which have significant meaning for epigenetic modification, post-transcriptional regulation of target genes, molecular interference, etc. The dysregulation of ncRNAs will mediate the pathogenesis of diverse human diseases, like cancer. Pancreatic cancer, as one of the most lethal malignancies in the digestive system that is hard to make a definite diagnosis at an early clinicopathological stage with a miserable prognosis. Therefore, the identification of potential and clinically applicable biomarker is momentous to improve the overall survival rate and positively ameliorate the prognosis of patients with pancreatic carcinoma. LncRNAs as one kind of ncRNAs exert multitudinous biological functions, and act as molecular sponges, relying on microRNA response elements (MREs) to competitively target microRNAs (miRNAs), thereby attenuating the degradation or inhibition of miRNAs to their own downstream protein-coding target genes, also thus regulating the initiation and progression of neoplasms. LncRNAs, which emerge aforementioned function are called competing endogenous RNAs (ceRNAs). Consequently, abundant research of lncRNAs as potential biomarkers is of critical significance for the molecular diagnosis, targeted therapy, as well as prognosis monitoring of pancreatic cancer.

## Facts

- Pancreatic cancer is a highly malignant tumor of digestive system. In addition to insidious onset and nontypical clinical manifestations, rapid invasion and metastasis of cancer cells result in most inpatients who lack surgical indications in the late clinical stage with poor prognosis.
- LncRNA has multiple functions about the regulation of gene expression, among which the molecular decoy function is the basis for lncRNA to serve as ceRNA and indirectly modulate the expression of target genes.
- Compared with the conventional RNA mechanism, the ceRNA mechanism exhibits “competitiveness” and “complexity”.
- The specific mechanism model of ceRNA is still controversial, as researchers have different conclusions on the contribution of intracellular abundance and affinity of ceRNA to the occurrence of ceRNA crosstalk as well as the possibility of ceRNA crosstalk under physiological and pathological conditions.
- Numerous experimental studies have found that different lncRNAs promote or suppress cancer through different ceRNA regulatory axes and downstream pathways. Therefore, these regulatory axes comprising lncRNA are expected to become new therapeutic targets for pancreatic cancer.

## Open Questions

- Traditional chemotherapy for pancreatic cancer has poor efficacy, and it is urgent to carry out clinical targeted therapy to improve the prognosis of patients. Therefore, it is of great significance to seek ideal targets.
- A large number of reports have confirmed that lncRNA has abnormal expression in various tumors when function as ceRNA, while there are relatively few relevant reports on human pancreatic cancer. Hence, it is crucial to clarify the specific role of lncRNA as ceRNA in the progression of pancreatic cancer and the corresponding mechanism.

## Introduction

Pancreatic cancer (PC), as a malignancy of the abdominal digestive system, has a critical high fatality rate with a 5-year survival rate of less than 10% [1]. Patients with PC have a median survival time of 3–6 months [2]. For the past few years, the incidence of PC has been on the rise, with an average of 216,000 new cases reported worldwide each year, resulting in more than 200,000 deaths a year [3]. PC has become one of the principal reasons for death in vast patients with solid tumors. Clinically, the onset of PC is insidious and progresses rapidly. Patients only have epigastric discomfort at the early stage, which leads to difficulty in timely detection. Therefore, a large proportion of patients with PC are already in the advanced stage when they quest treatment for cachexia. At present, there are few mature clinical detection methods for PC. Imaging examination and immunological examination represented by CA19-9 have become typical inspection methods, but the missed detection rate is still high, while early diagnosis and etiological diagnosis cannot be carried out. Although the comprehensive treatment methods for PC including systemic chemotherapy have been improved, due to the high invasiveness, rapid migration of PC, as well as other malignant biological behaviors, it is still hard to guarantee the quality of patients’ life in the later period with tragic surgical resection rate and high postoperative recurrence rate. Therefore, the therapeutic effect and prognosis of pancreatic ductal adenocarcinoma as the main pathological pattern are extremely poor at the present stage.

Over the past few decades, various clinical trials of targeted therapies for PC have been conducted, and some of them have unfolded promising results in subgroups of patients with PC. The experience of these clinical trials, whether successful or unsuccessful, will help refine targeted therapies for PC over the next few years [4]. Thus, there is an imperative need to search latent biomarkers for early diagnosis, radical treatment, and improvement of prognosis for patients with PC. LncRNAs, as a set of transcripts with regulatory functions, have been identified to be involved in the biogenesis of multifarious neoplasms [5], while tumor phenotype can also be altered by regulating their expression [6]. Some lncRNAs have been thoroughly studied, like H19, Metastasis associated lung adenocarcinoma transcript 1 (MALAT1), or HOX transcript antisense intergenic RNA (HOTAIR), are considered to be pan-cancer markers involving multiple diverse malignancy tissues [7]. As an oncogene in PC, the expression level of lncRNA HOTAIR was significantly correlated with susceptibility of PC [8], as well as the propagation and migration of PC cells [9]. MALAT1 was firstly recognized in non-small-cell lung cancer [10]. Nevertheless, upregulated expression of MALAT1 has presented in multiple carcinoma tissues comprising PC [11]. In PC, MALAT1, which functions as an oncogene accelerated cell proliferation, invasion, and migration [12]. Similarly, H19 was markedly overexpressed in PC tissues, the level of expression was positively correlated with histopathological grade and malignant phenotype including aggressiveness [13]. Furthermore, lncRNAs are widely detected in body fluids consisting of blood, saliva, urine, and even pancreatic juice [2]. Therefore, as a novel biomarker closely related to tumor diagnosis, targeted therapy, and improvement of patient prognosis, it is particularly necessary to further explore the function as well as the corresponding mechanism of lncRNA in PC.

## Long non-coding RNAs

As is well-known, despite more than 75% of the human genome partakes in transcription, only 2% of it possesses protein-coding function [14]. Therefore, major transcripts, containing ncRNAs, are non-coding genome sequences [14]. LncRNAs are a category of ncRNA transcripts that lack the function of encoding peptides or proteins. Nonetheless, lncRNAs are essential for the proper functioning of cellular processes owing to the modulation of gene expression at disparate levels [15].

Although certain types of lncRNAs, which have been well researched are no more a mystery, the definition and classification of lncRNAs maintains indistinct on account of the precise mechanisms and specific signaling about these molecules, which have not been absolutely illuminated.

The length of lncRNAs is >200 nucleotides, most between 1000 and 10000 nucleotides. They were incipiently thought to be a form of transcriptional “trash” or “noise” synthesized by RNA polymerase II, which were believed to be incapable of modulating any biological behavior [16]. According to their different characteristics, lncRNAs can be divided into diverse species (Table 1).

**Table 1:** The classification of lncRNAs.

Taxonomy	Species	Characteristics	References
Genomic origins	Sense/antisense	Sense or antisense lncRNA locates within or overlaps with the exons of the associated protein-coding gene on the same or opposite strand, while antisense lncRNA transcribes in the opposite direction of protein-coding gene	[131]
	Bidirectional	Bidirectional lncRNA locates nearby the promoter of the associated protein-coding gene and transcribes in the opposite direction	[131]
	Intronic	Intronic lncRNA arises from long introns and transcribes from inside of an intron of the associated protein-coding gene	[131]
	Intergenic	Intergenic lncRNA originates from intergenic segment of two protein-coding genes	[131]
Function	rRNA	A major structural component of the ribosome that interacts with specific mRNA sequences. Prokaryotic rRNAs are 5 S^a^, 16 S, and 23 S, while eukaryotic rRNAs are 5 S, 5.8 s, 18 S, and 28 S, of which 16 S, 23 S, 18 S, and 28 S are long non-coding RNAs	[132]
	cRNA	cRNAs interact with chromatin by the recruitment of the polycomb repressive complex (PRC) mostly. PRC induces chromatin modification, which leads to epigenetic gene silencing	[133, 134]
	eRNA	eRNAs can increase the expression of target genes in cis by increasing the strength of the enhancer-promoter looping or impede the binding of negative elongation factors (NELFs) to the promoter thereby alleviating transcriptional repression	[135–137]
	SINEUP	SINEUPs are modular antisense lncRNAs with an inverted SINEB2 sequence and a small complementarity sequence of the targeted mRNA, which up-regulate the translation of mRNAs in a gene-specific manner without affecting gene expression	[138–140]
	ceRNA	ceRNAs as miRNA sponges compete with mRNAs for miRNA binding, thus impairing the biological activity of miRNA	[47, 141]
Subcellular localization	Nuclear lncRNA	Most of the nuclear lncRNAs are regulators of transcription, and they can both enhance or silence the transcription of genes by recruiting transcription factors or by acting as decoy impeding the binding of transcription factors to DNA	[142]
	Cytoplasmic lncRNA	Cytoplasmic lncRNAs are more commonly involved in post-transcriptional regulation	[142]
	Mitochondrial lncRNA	The light strand of mitochondrial DNA codes one subunit of nicotinamide adenine dinucleotide dehydrogenase (ND), 8 tRNAs, and 3 lncRNAs, while these lncRNAs regulate ND5 (NADH dehydrogenase subunit 5), ND6 (NADH dehydrogenase subunit 6) and CYTB (cytochrome b) throughout complementary binding of respective RNAs. Moreover, mitochondrial DNA allows the synthesis of two lncRNAs (SncmtRNA and ASncmtRNA) related to cell proliferation or tumor suppression, while LIPCAR is associated with the risk of heart failure	[143–146]

*rRNA* ribosomal RNA, *cRNA* chromosomal RNA, *eRNA* enhancer RNA, *ceRNA* competing endogenous RNA, *SncmtRNA* sense noncoding mitochondrial RNA, *ASncmtRNA* antisense noncoding mitochondrial RNA, *LIPCAR* long intergenic noncoding RNA predicting cardiac remodeling.

^a^Sedimentation coefficient.

Dysregulation of lncRNAs has been convinced of being closely connected with the pathogenesis of malignancies. Hence, it is crucial to inquire about the complicated molecular biological functions of lncRNAs. LncRNA molecules have been identified to possess four disparate functional archetypes, acting as signals, decoys, guides, as well as scaffolds respectively [17].

The transcription of lncRNAs occurs at a precise time and spot to integrate developmental signals, which uncovers the intracellular environment, or reacts to various irritants, indicating that lncRNAs are capable of serving as molecular signals and markers about important biological incidents [17]. In this archetype, lincRNA-p21 exerts a specific effect on triggering apoptosis as a transcriptional target of p53 [18]. For the second archetype, lncRNAs work as molecular decoys, which bind and titrate away proteins or RNA targets, thereby exerting negative effect generally. Some lncRNAs act as “microRNA-sponges” to compete with mRNAs for microRNA binding, which reduce recognition rate and biological activity of microRNA consequently, while modulate the progression of cancers, such as H19 [19], HOTAIR [20], MALAT1 [21], and XIST [22]. What’s more, the other molecular function of lncRNAs is a guide. LncRNAs bind to proteins and then target the synthetic complex to a specific target, where it interacts straightway with DNA or RNA via base pairing [17, 23]. Thus, guided lncRNAs can activate or inhibit the subsequent expression of their target genes by modification at transcriptional level. For instance, the adaptor protein WDR5 in the WDR5/MLL complex can target HOXA by immediately binding to lncRNA HOTTIP, which acts as a guide at this time, thereby increasing the H3 lysine 4 trimethylation of HOXA cluster and activating its transcription [17]. In addition to the above three archetypes, lncRNAs also function as scaffolds. LncRNAs serve as central platforms for the assembly of effector molecules, while disparate lncRNA regions simultaneously combine diverse effector molecules, leading to activation or restraint of transcription [17]. For example, Kcnq1ot1, which stimulates H3K27me3 and H3K9me3 by synchronously binding PRC2 as well as G9a [24].

In summary, RNA-binding domains, DNA-binding domains, and protein-binding domains all belong to the functional domains of lncRNAs [25]. In addition, the various archetypes mentioned above do not exist in isolation but can coexist in the process where lncRNAs play a regulatory role. It is precisely such diverse lncRNA complexes formed by various molecular interactions that exert their powerful gene regulatory functions by regulating their localization and stabilization or conducting biological modification.

## Competing endogenous RNAs

### Definition and hypothesis of ceRNAs

Competing endogenous RNAs (ceRNAs) refer to a class of coding or non-coding RNAs which can competitively bind microRNAs (miRNAs) and sequester miRNAs from their original target transcripts so as to avoid the degradation or expression inhibition of target transcripts induced by miRNAs at the post-transcriptional and translational levels. In such a molecular biological model, miRNAs thus play an indispensable role.

**Fig. 1:**
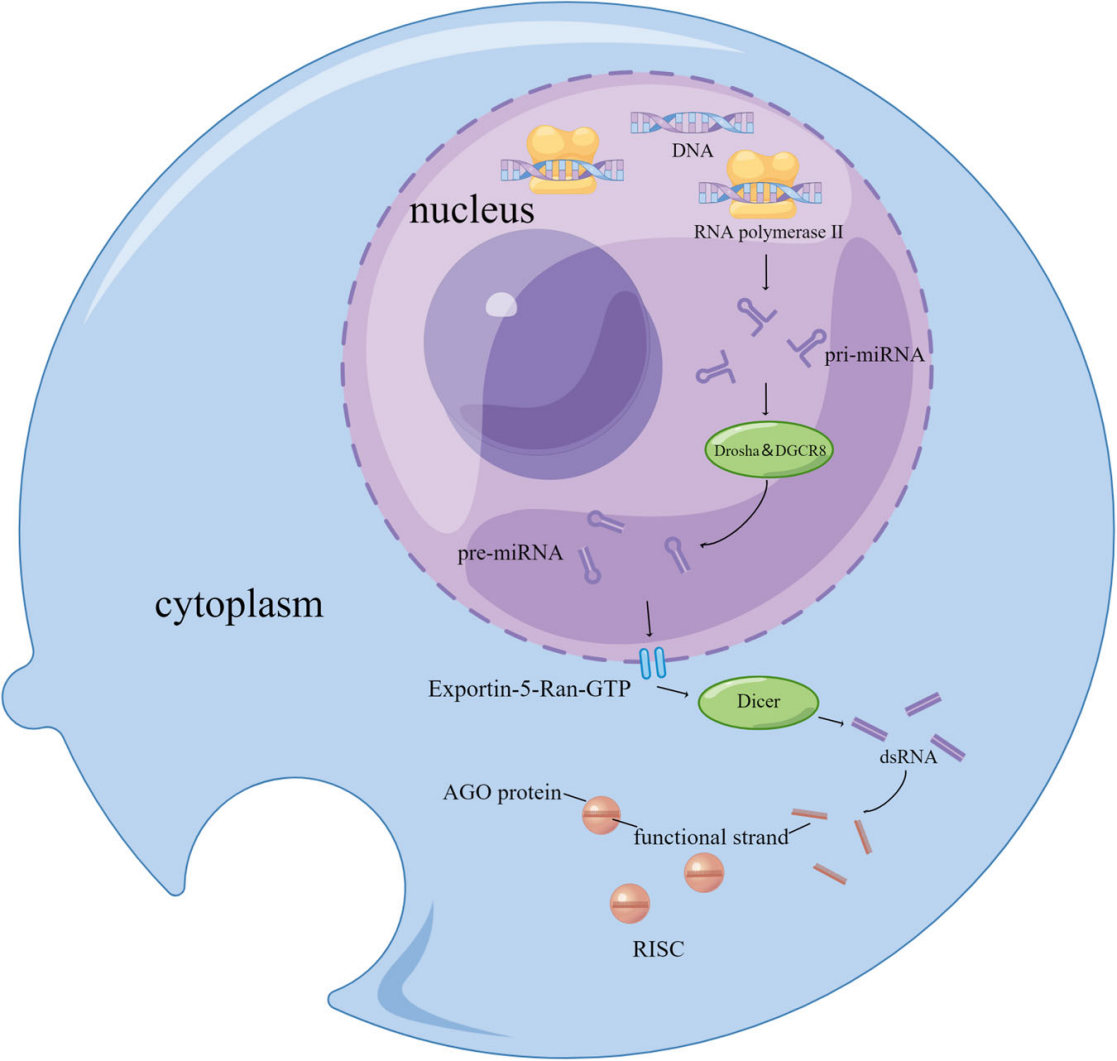
Formation process of mature miRNA and RISC. Cap-shaped poly-adenylated pri-miRNAs are encoded in the nucleus by RNA polymerase II, which are then cleaved by Drosha and DGCR8 to generate hairpin-shaped pre-miRNAs. The pre-miRNAs are subsequently exported to the cytoplasm through the Exportin-5 channel and cleaved by another ribonuclease, Dicer, to produce short double-stranded RNA (dsRNA) fragments whose isolated functional chains subsequently participate in AGO protein and eventually form RNA-induced silencing complex (RISC). (By Figdraw (www.figdraw.com)).

Mature miRNAs are short, single-stranded non-coding RNAs that are 19–25 nucleotides in length, accounting for 1–5% of the human genome. Approximately 28,000 mature miRNAs have been recognized, while 60% of human protein-coding transcripts are evolutionarily conserved targets for miRNAs [26–28]. The process of mature miRNAs biogenesis initiates with transcription of a nucleotide sequence from intergenic or intron-coding regions via RNA polymerase II, forming cap-shaped, poly-adenylated transcripts named primary-microRNAs (pri-miRNAs). In rapid sequence, pri-miRNA is then cleaved by the microprocessor complex (Drosha and DGCR8), a hairpin-shaped intermediate consisting of 70–100 nucleotides is produced, called precursor-miRNA (pre-miRNA) [29, 30]. The pre-miRNA subsequently traverses the karyotheca into the cytoplasm through the Exportin-5-Ran-GTP channel, where it is ulteriorly cleaved by another ribonuclease, Dicer, resulting in short, double-stranded RNA fragments whose strands separate soon afterwards, while the functional strand is then involved in an Argonaute (AGO) protein, thereby generating the RNA-induced silencing complex (RISC), which exerts biological function as the last authentic effector molecule [31] (Fig. 1).

For target transcripts, the biological function of miRNAs includes two aspects. One is that miRNA induces the cleavage and degradation of target mRNA through complete base pairing with the 3′ untranslated region (3′ UTR) of mRNA; the other is that miRNA cannot perfectly complement with target mRNA, and its effect is only to restrain the translation of mRNA. Regardless of the interaction between miRNA and target mRNA, the consistent end result is that the protein expression products of target mRNA are reduced. Therefore, miRNAs have a pivotal effect on post-transcriptional regulation as modulators. Furthermore, miRNAs have been implicated in multifarious physiological and pathological regulatory courses, while being associated with stress responses and many human diseases, including tumors [32–34].

According to the experiments and observations so far, we believe that the inhibition function of miRNA itself is also regulated by the target transcripts containing miRNA binding sites, that is, miRNA does not play a one-way inhibitory function, but interacts with ceRNA in addition. Currently, mRNA does not just play the role of protein encoding and passively accepts miRNA-mediated regulation. Instead, it is seen as a member of the intracellular ceRNA crosstalk, actively interacting with miRNA. The centralized regulation of ceRNAs and their interaction with miRNAs is achieved by recognizing miRNA response elements (MREs) of target transcripts [35, 36]. MREs, typically 2–8 nucleotides, are sited in coding sequences (CDS), 5′ untranslated regions (5′ UTRs), and mostly 3′ UTRs of several RNA subsets including transcribed pseudogenes, lncRNAs, circular RNAs (circRNAs), and mRNAs [37–39]. In the ceRNA hypothesis, non-coding RNAs and mRNAs with non-coding properties combine into a functional complex, forming a multilevel and trans-regulatory ceRNA network (ceRNET) on the transcriptome [40], in which competition and interaction occur among all ceRNA subgroups. Together, they elucidate the underlying molecular mechanisms and post-transcriptional-layered interpretations of pathogenesis and the development of vast disordered conditions like cancer [40]. In recent years, some have suggested extending the concept of ceRNA to any RNA crosstalk surrounding common regulators [41], while others have also proposed the concept of “ceRNome”, which refers to the integration of interrelated RNA molecules in a comprehensive cellular environment [42], suggesting ceRNA crosstalk does not occur independently. Rather, most of them coexist in a monolithic post-transcriptional context.

Back in 2007, Ebert et al. demonstrated that artificially expressed mRNAs containing a large proportion of miRNA binding sites with high-affinity in mammalian cells can alter miRNA-mediated inhibition of targets [43]. In the same year, lncRNA IPS1 was realized in *Arabidopsis thaliana* by Franco-Zorrilla and others, which sequesters phosphate starvation-induced miR-399 and increases the stability and abundance of target PHO2 subsequently [44].

Later in 2010, it was reported that *Herpesvirus saimiri* transformed T cells expressed a non-coding RNA called H. saimiri U-rich RNAs (HSURs), which were associated with decreased miR-27 activity and incremental intracellular FOXO1 levels [45]. Meanwhile, the first mammalian ceRNA, PTENP1, was confirmed experimentally, which is a pseudogene transcription product that shares multiple conserved miRNA binding sites with tumor suppressor gene PTEN, thus regulating the mRNA level and protein abundance of PTEN in a miRNA-dependent manner [46]. Poliseno et al. found that selective deletion of PTENP1 promoted the occurrence and development of human cancer, and further proved its inhibitory effect on tumor cell proliferation [46]. The above evidences had laid a solid foundation for the ceRNA hypothesis, which was eventually officially presented by Pandolfi et al in 2011 [47].

### Existence mode of ceRNAs

In the human genome, only about 2% protein-coding genes, while about 95% were incipiently regarded as meaningless evolutionarily remnants, and referred to as “junk DNA” [35]. With the deepening of the research progress, most of this “junk DNA” radiates vitality, and is transcribed in diverse spatiotemporal circumstances. Ultimately, in addition to the rRNA and tRNA, most ncRNAs, which are implicated in the modulation of gene expression, especially in the transcriptional and post-transcriptional levels, and participate in a variety of signaling pathways. As mentioned above, miRNA has a central effect on the ceRNA regulatory network, while ceRNA is the fine-tuning regulator of the effects generated by the whole regulatory network. The constituent modules of ceRNAs are mostly composed of mRNAs and ncRNAs including transcribed pseudogenes, lncRNAs, and circRNAs. Thus, the ncRNA/miRNA/mRNA axis is formed.

Pseudogenes are remnants of parental genes, which have lost the coding function of full-length functional proteins along with replication and mutation in the process of evolution [48, 49]. Vast evidences indicate that pseudogenes are a crucial part of the intricate, multi‐layer regulatory network regulating gene expression [50]. Pseudogenes can be classified into disparate categories, manifesting malignancy specificity, pedigree specificity, and widely expressed pseudogenes respectively [51], which also indicates that pseudogenes play a role in tumor characterization and supply a promising prospect for diagnosis and therapy. Pseudogene transcription products include non-coding RNA and antisense RNA [52], which can generate anti-cancer and pro-cancer effects through the ceRNA regulatory network. Typically, pseudogene PTENP1, as a counterpart of tumor suppressor PTEN, competitively binds to miR-21 through shared MREs while alleviates the inhibitory effect of miRNA on PTEN, thus upregulating PTEN expression while playing a suppressive role [53].

As stated above on the molecular function of lncRNA, lncRNA plays an important role in ceRNA crosstalk as a molecular decoy. H19 has an overexpression during embryonic development as well as postnatal growth but is completely inhibited during adulthood. Elevated H19 expression has been identified from research about many malignancies, which is also related to genomic instability [54]. Initially, the carcinogenic function of H19 was thought to be mediated by direct or indirect targeted inhibition of miR-675 [55]. However, there is accumulated evidence that H19 also plays an additional role as miRNA decoy in tumorigenesis and promotes the malignant phenotype of tumors through epithelial-mesenchymal transition (EMT). H19 facilitates metastasis of bladder cancer and pancreatic cancer through miR-29b-3p/DNMT3B (DNA methyltransferase 3β) axis and let-7/HMGA2 axis respectively [56, 57]. Current information on MALAT1 mostly emphasizes relevant carcinogenic effect on multiple malignancies. Numerous researches already elucidated MALAT1 induces proliferation, invasion and migration through miRNA-mediated manner in colorectal cancer (CRC), breast cancer, gallbladder cancer, non-small cell lung cancer (NSCLC), and oral squamous cell carcinoma (OSCC) [58–62].

Circular RNAs (circRNAs) are generated by nearly 20% of functional genes and are widely expressed in mammalian cells [63]. The self-circularization structure of circRNA relies on the covalent binding of the 3′ and 5′ ends after “backsplicing” [64]. Compared with the linear structure, it has higher stability and can resist exonuclease-induced degradation and miRNA-mediated repression due to the lack of free ends [64]. In addition, extensive evidences have revealed that circRNA plays a pivotal role in dominant intracellular localization of ceRNA in malignancy progression [65], and circRNA’s unique stability makes it ideal circulating markers in body fluids such as plasma, serum, or saliva [66, 67]. As one of the first functionally characterized circRNAs, Cerebellar degeneration-related protein 1 antisense RNA (CDR1as) participates in the formation of the CDR1as/miR-7 axis, namely ciRS-7 (circRNA sponge for miR-7) [68], through the ceRNA regulatory mechanism, and exerts a carcinogenic effect on the progression of HCC, CRC, NSCLC, as well as gastric cancer [69–72]. Additionally, circHIPK3 (Homeodomain Interacting Protein Kinase 3) and circPVT1 demonstrate extensive carcinogenic capacity by sequestering miR-124/miR-7 and let-7 respectively [73–75]. On the contrary, circ-ITCH originates from few exons of ITCH, a ubiquitin-ligase E3, which plays a tumor suppressor by facilitating ubiquitin degradation of DVL2 (Dishevelled segment polarity protein 2) to repress typical Wnt signaling pathway [76]. It is validated that circ-ITCH resists miR-7, miR-17, and miR-214, while upregulating ITCH by blocking the Wnt/β-catenin pathway, thereby impeding the growth of NSCLC and esophageal squamous cell carcinoma (ESCC) [77, 78]. Similarly, circ-ITCH upregulates p21 and PTEN by sequestering miR-7 and miR-224 and suppresses the malignant phenotype of bladder cancer [79].

### Functional mechanism of ceRNAs

**Fig. 2:**
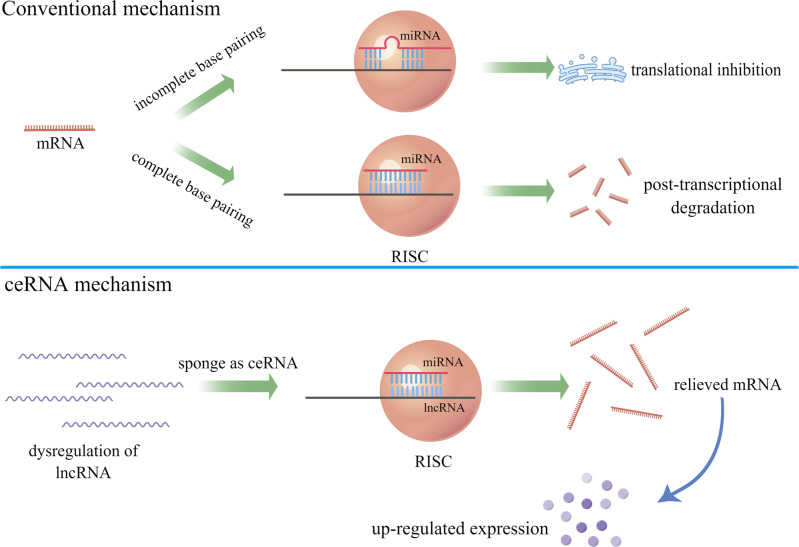
Differences between conventional and ceRNA mechanisms. In conventional mechanism, RISCs induce mRNAs degradation through complete base pairing, while the translation of mRNAs is blocked by RISCs through incomplete base pairing. In ceRNA mechanism, lncRNAs sponge miRNAs by serving as ceRNAs to relieve mRNAs while further improve the abundance of expressed products. (By Figdraw (www.figdraw.com)).

More than a decade ago, Seitz believed that the vast majority of transcripts with MREs, which are called “miRNA sponges” in functional classification can function as effective blocker of miRNA, thereby regulating the role of miRNA via contending with endogenous mRNAs for shared miRNA binding sites [80]. Compared with conventional RNA logic, “competition” and “interaction” have become the core of the theory after the new concept of ceRNA was proposed in 2011 [47]. Therefore, the complicacy and relativity of the ceRNA regulatory network are of great significance. Theoretically, when other ceRNAs containing shared MREs are involved, miRNA no longer plays a one-way role in degrading or inhibiting the expression of its downstream mRNA but forms a bidirectional interaction between ceRNA and miRNA. The resulting effects include: On the one hand, the molecular level of miRNA may be reduced, while the availability and activity of miRNA might be impaired. On the other hand, the intracellular abundance of different ceRNAs can be adjusted mutually, that is to say, the increase of transcription level on one side would alleviate the repression induced by miRNA on the other side, thus indirectly regulating gene expression [40] (Fig. 2).

### Molecular bases and models of ceRNA crosstalk

In addition to the MREs mentioned above, there are also RNA-binding proteins (RBPs) binding sites in the ceRNA sequence [40]. RBPs can directly occupy specific binding sites or indirectly change the affinity of ceRNA to miRNA through the rearrangement of ceRNA secondary structure, thus altering the degree of interaction between ceRNA and miRNA.

What’s more, there are still subtle differences in nucleotide components in shared MREs due to single nucleotide polymorphism (SNP) [40]. And it is confirmed that miRNAs with shortened 3’ UTRs are formed after alternative splicing [81]. Both effects are ultimately embodied in the altered affinity between miRNA and ceRNA with MRE.

In view of the stoichiometric relationship among miRNAs and their targets, Bosson et al. proposed that miRNAs preferentially bind to mRNAs with high-affinity target sites, compared with more abundant and lower affinity sites [82]. This hierarchy can effectively reduce the number of miRNA binding sites and even the number of target transcripts. Therefore, in the ceRNA network, it is easy to lead to the derepression of other miRNA-targeted transcripts for ceRNAs with high-affinity miRNA binding sites as the number of such sites is decreased. In addition, studies have indicated that in this hierarchical model, under the condition of low or intermediate miRNA:target ratio, ceRNA abundance, even at the physiological level, adequate miRNA binding sites are able to be supplied to relieve miRNA-mediated repression on other target transcripts [83].

Subsequently, Denzler et al. proposed another model, suggesting that the binding between miRNA and the target transcript has little correlation with the affinity of the miRNA binding sites [84]. Therefore, only a mass of miRNA binding sites, which come from ceRNAs can alleviate inhibitory effect by miRNA. Denzler et al. believed that whatever transcript or global variation in transcription abundance cannot contribute enough additional binding sites to reverse the target inhibition by miRNA, and thus inferred that ceRNA crosstalk was impossible when transcription abundance was within the physiological range [84, 85] (Table 2).

**Table 2:** Differences between two ceRNA crosstalk models.

	Hierarchical model	Non-hierarchical model
Presenters	Bosson et al.	Denzler et al.
Key points	miRNAs mainly bind to high-affinity target sites	The binding of miRNAs to target sites is independent of target site affinity, but target the transcriptome extensively and evenly
Determinants of ceRNA crosstalk	Ratio of miRNA abundance to higher affinity binding sites on the target transcripts	Abundance of miRNA binding sites in the transcriptome
Extended conclusions	ceRNA crosstalk can occur even under physiological conditions when the miRNA:target ratio is low	ceRNA crosstalk is difficult to occur under physiological conditions
References	[82]	[85]

## LncRNAs as ceRNAs in the carcinogenesis and development of pancreatic cancer

LncRNA, as a member of many categories of ceRNAs, achieves epigenetic modification and pivotal post-transcriptional regulation through the mechanism of ceRNA network. Nowadays, accumulating evidences indicate that in pathological states such as neoplasm, intracellular lncRNA abundance is sufficient to trigger ceRNA crosstalk, and lncRNA can sponge miRNA for a long time through incomplete complementary binding between MRE and miRNA, thus changing the activity and availability of miRNA, while regulating the expression of downstream target genes. Therefore, alterations in the affinity of ceRNA to miRNA or its own abundance can activate or impede downstream signals, thereby promoting or repressing the carcinogenesis and malignant phenotype of cancer. Numerous experimental data have confirmed that the identified lncRNA-mediated ceRNA regulatory network, namely lncRNA/miRNA/mRNA axis, plays a role of promoting or suppressing cancer in the oncogenesis and progression of pancreatic cancer (PC) via multiple cell functions. In addition, according to existing reports, like miRNA, aberrant expression of lncRNA also has clinical applicability, which has potential to work as the biomarker for precancer diagnosis and prognosis of human breast, liver, colorectum, and lung malignancies [86–89].

### LncRNAs act as ceRNAs to promote pancreatic cancer

Small nuclear RNA host gene 12 (SNHG12) has 675 nucleotides, which orientates at chromatin 1 and has been reported to militate for the progression of TNBC, gastric carcinoma, prostate carcinoma, CRC as well as glioma [90–94]. Recently, Cao W et al. inquired the pathological relevance between lncRNA SNHG12 and PC, while uncovered potential mechanisms [95]. It was confirmed that lncRNA SNHG12 expression quantity in PC tissues and metastatic PC tissues was augmented compared with para-cancerous tissues as well as non-metastatic PC tissues [95]. In addition, the result of qRT-PCR showed that the quantity of SNHG12 was augmented in four PC cell lines (BxPC3, CAPAN1, PANC1, SW1990) compared with human pancreatic ductal epithelial cell line (HPDE6) [95]. Elucidated by cell function experiments that si-SNHG12 restricted PC capacity of propagation and aggression, while restrained epithelial-mesenchymal transition (EMT) [95]. Therefore, SNHG12 had a carcinogenic effect on PC progression. More importantly, intracellular overexpression of SNHG12 led miR-320b decreased while enhancing EMT. Inversely, overexpression of miR-320b reduced intracellular abundance of SNHG12 [95]. Hence, SNHG12 had a negative correlation with miR-320b in PC, further revealing the pivotal role of SNHG12/miR-320b/EMT axis in PC development. Thus, SNHG12/miR-320b may work as a target for PC treatment.

The antisense of KTN1, RNA1 (KTN1-AS1), as a lncRNA, has been found to function as an oncogenic gene in HNSCC and HCC [96, 97]. At present, Zhang ZB et al. unfolded the function of KTN1-AS1 in PC and further analyzed the relevance between KTN1-AS1 and clinicopathological information of PC patients [98]. Overexpression of KTN1-AS1 in PC samples implied a golden diagnostic value. Additionally, the quantity of KTN1-AS1 had a positive correlation with clinicopathological stage, histopathological grading of PC [98]. Knock-down of KTN1-AS1 induced impaired propagation and aggressiveness ability while elevated apoptosis rate, but it showed opposite effect when KTN1-AS1 was overexpressed [98]. Dual luciferase reporter (DLR) assay, RNA-immunoprecipitation (RIP) assay, as well as functional tests confirmed upregulated miR-23b-3p lowered abundance of KTN1-AS1 and HMGB2 in PC cells, ulteriorly suppressed cell propagation and aggressiveness, but enhanced apoptosis [98]. Besides, sh-KTN1-AS1 by transfection reversed the repressive impact of miR-23b-3p-mimics on HMGB2, implying that KTN1-AS1 could sponge miR-23b-3p to indirectly modulate HMGB2 [98]. Thus, it can be concluded that KTN1-AS1 contributed to pancreatic adenocarcinoma development via miR-23b-3p/HMGB2.

LncRNA PC-esterase domain containing 1B antisense RNA 1 (PCED1B-AS1) has been recognized in the modulation about multiple disease progression like active tuberculosis, glioma, and luminal-B breast cancer [99–101]. Zhang Y et al. had demonstrated the biological function, potential mechanism, and clinical value in PC by experiment research [102]. What had been confirmed was that PCED1B-AS1 had a positive correlation with advanced Tumor-Node-Metastasis (TNM) stage with an elevated expression in PC [102]. PCED1B-AS1 exerted oncogenic effect on the phenotype of PC such as propagation, aggressiveness, as well as EMT in vitro using Cell Counting Kit-8, EdU staining, and Transwell assays respectively [102]. By means of bioinformatics analysis and verification experiments of gene regulatory relationship, PCED1B-AS1 was clarified to sponge miR-411-3p, serving as a ceRNA mechanistically, contributing to the up-regulation of hypoxia-inducible factor-1α (HIF-1α) [102]. Therefore, the PCED1B-AS1/miR-411-3p/HIF-1α axis, which possesses the key impact on PC progression, providing bright prospects for PC diagnosis and treatment.

LncRNA HLA complex group 11 (HCG11) has been identified in various malignant tumors. According to recent studies, HCG11 suppressed apoptosis to accelerate HCC progression in addition to facilitate neoplasm germination and motility in GC via miR-1276/CTNNB1 axis [103, 104]. However, in glioma and laryngeal carcinoma, HCG11 was verified to exert inhibiting effects on the development of malignancies via different ceRNA regulatory network [105, 106]. So, it was unfolded that HCG11 might exert a dual effect on diverse malignancies. In pancreatic cancer, Xu J et al. illustrated that HCG11 competitively targeted miR-579-3p to augment MDM2 expression, thereby activating the downstream Notch/Hes1 signaling pathway to accelerate the progression [3]. Moreover, HCG11 and MDM2 were discovered to reverse the inhibiting effects of miR-579-3p on cancer progression, including inhibiting germination, enhancing cycle retardation, increasing death rate, while repressing motility by rescue assays [3]. Through animal model, tumor size and weight were measured to evaluate the tumor-bearing effect in nude mice, which further proved the oncogenic role of HCG11 in vivo [3].

Dysregulated TP73-AS1 has been recognized in multifarious malignancies, like glioma, HCC, as well as NSCLC [107–109]. Besides, overexpression of TP73-AS1 was significantly related to miserable prognosis in patients with osteosrcom and CRC [110, 111]. In order to ulteriorly research the underlying mechanism of TP73-AS1 in PC, DLR testified that TP73-AS1 was negatively correlated with miR-128-3p in the case that TP73-AS1 was forecasted to target miR-128-3p by bioinformatics analysis [112]. Similarly, qRT-PCR and western blotting detected that intracellular abundance and translational products of target gene GOLM1 were decreased due to the overexpression of miR-128-3p [112]. Hence, TP73-AS1-mediated regulatory network around miR-128-3p exerted vital impact on PC progression. The above molecular mechanism demonstrated that TP73-AS1 could work as a prognosis biomarker for PC patients.

In addition to the above regulatory axis, there are many other lncRNA-mediated ceRNA regulatory networks that exert disparate and essential impacts on PC pathogenesis and development, like: THAP9-AS1/miR-484/YAP, MIR31HG/miR-193b, MALAT1/miR-217/KRAS axis regulate the growth and survival of tumor cells [113–115]. Furthermore, AFAP1-AS1 targets miR-384 and upregulates downstream ACVR1 to induce pancreatic cancer stem cell maintenance [116]. LINC00511/miR-29b-3p/VEGFA axis modulates angiogenesis in PC [117].

### LncRNAs act as ceRNAs to suppress pancreatic cancer

The lncRNA DLEU2L (deleted in lymphocytic leukemia 2-like) is located on chromosome 1p31.3, which functions as a repressor in PC in vitro as well as in vivo [118]. The studies had verified that DLEU2L sponged miR-210-3p through competing with BRCA2 via ceRNA mechanism [118]. In previous research, miR-210-3p worked as an oncogene, had a positive correlation with malignant biological behaviors in PC cells, including proliferation, invasion, and migration [119]. These effects were closely associated with activation of downstream AKT/mTOR signaling pathway, which was involved in the potential mechanism of multifarious malignancies progression, like autophagy, chemoresistance, and the Warburg effect [120–122]. As a matter of fact, the drug resistance of tumors was closely related to metabolic reprogramming induced by the Warburg effect [123]. As was known that gemcitabine was a chemotherapy agent for multiple malignancies including PC, and during the progress where DNA replication arrest was converted into double-strand break induced by gemcitabine, BRCA2 was recruited to inhibit DNA replication and damage repair, which further promoted gemcitabine cytotoxicity and ultimately led to cell death [124]. Therefore, over-expressed DLEU2L targeted miR-210-3p, reduced its biological activity and simultaneously upregulated intracellular BRCA2 level, mTOR phosphorylation was inhibited, thus decreasing the potential chemotherapy resistance of gemcitabine in PC treatment and enhancing its cytotoxic effect.

It was reported that a novel lncRNA, LINC01111, was markedly downregulated in PC tissues and plasma of PC patients acting as a tumor suppressor [125]. It was unfolded that LINC01111 impaired the tumorigenesis, germination, and migration via functional experiments in vitro as well as nude-mouse xenograft tumor model in vivo [125]. Mechanistically, it was found that overexpression of LINC01111 upregulated DUSP1 level through sponging miR-3924, leading to the impediment of SAPK phosphorylation as well as the deactivation of the SAPK/JNK signaling pathway in PC cells, thus suppressing PC aggressiveness [125]. In general, the above information disclosed LINC01111 may serve as a diagnostic and prognostic biomarker while the newfound LINC01111/miR-3924/DUSP1 axis might work as an underlying curative target in the near future.

Dysregulated lncRNA growth arrest-specific 5 (GAS5) was reported to be involved in tumor propagation, metastasis, as well as EMT in osteosarcoma [126]. Consistently, the consequences indicated that GAS5 had a low expression in PC tissues and cell lines, while upregulated GAS5 repressed cell propagation, aggressiveness, migration, as well as gemcitabine resistance [127]. So, GAS5 exerted tumor-suppressive effects in PC. Previous research discovered that miR-221 facilitated the propagation of CAPAN-2 PC cell line by targeting PTEN-Akt [128], which suggested that miR-221 might exert a carcinogenic impact on the development of PC. Conformably, recent experiments showed that miR-221 facilitated PC cell propagation, metastasis, as well as chemoresistance by accelerating the EMT in addition to cancer stem cell (CSC) accumulation [127]. In addition, it was found that the expression of SOCS3 led to the inactivation of JAK2/STAT3 signaling, which induced CSC properties [129, 130]. Bioinformatics analysis and experiments confirmed the interplay among GAS5, miR-221, as well as SOCS3. GAS5 curtailed the repressive effect on target transcript SOCS3 through sponging miR-221, thereby inhibiting the malignant biological behaviors of PC cells [127]. Hence, modulating the GAS5/miR-221/SOCS3 axis could be a promising treatment strategy for PC.

## Conclusions

As a highly malignant tumor of digestive system, pancreatic cancer has the characteristics of insidious onset, rapid disease development, poor therapeutic effect, and undesirable prognosis. At present, exploring biomarkers and therapeutic targets for early diagnosis with clinical applicability has become a research hotspot.

The ceRNA hypothesis adds a potential mechanism about the regulation of gene expression in tumorigenesis, while further supplies a new opportunity for remedying various human malignancies. In the ceRNA networks, ncRNAs as well as protein-coding RNAs is closely linked through interaction, thus breaking the conventional RNA logic. LncRNA, as a member of the composition of ceRNAs, plays the function of molecular decoy or sponge by virtue of the competitive combination between MRE and miRNA. More importantly, lncRNAs function as ceRNAs further regulate the intracellular abundance and expression product quantity of downstream target genes at the post-transcriptional and translational layers.

As discussed in this review, compared with normal pancreatic tissues, the expression of lncRNA in pancreatic cancer is markedly different, while it is also vitally correlated with tumor stage and survival prognosis. However, different lncRNAs may exhibit different expression levels in pancreatic cancer, thus playing widely diverse roles as oncogenes or tumor suppressors in the progression of pancreatic cancer. In recent experiments conducted by researchers, lncRNAs act as ceRNAs, and modulate many malignant biological characteristics including cell proliferation, invasion, metastasis, and chemotherapy resistance through the Warburg effect, EMT, cancer stem cell maintenance, and other mechanisms in vitro, while have been verified in vivo by animal experiments (Table 3).

**Table 3:** lncRNAs function as ceRNAs in pancreatic cancer.

LncRNA	Role in PC	miRNA	Target	Function and mechanism	References
SNHG12	Oncogenic	miR-320b	ZEB	Promotes cell proliferation, invasion, and EMT	[95]
KTN1-AS1	Oncogenic	miR-23b-3p	HMGB2	Enhances proliferation and invasion ability, while inhibits apoptosis	[98]
PCED1B-AS1	Oncogenic	miR-411-3p	HIF-1α	Promotes cell proliferation, invasion as well as EMT	[102]
HCG11	Oncogenic	miR-579-3p	MDM2	Accelerates cell growth, enhances mobility, and inhibits apoptosis by activating the Notch/Hes1 signaling	[3]
TP73-AS1	Oncogenic	miR-128-3p	GOLM1	Promotes pancreatic cancer growth and metastasis	[112]
THAP9-AS1	Oncogenic	miR-484	YAP	Positive regulation of tumor growth and survival	[113]
MIR31HG	Oncogenic	miR-193b	–	Positive regulation of tumor growth and survival	[114]
MALAT1	Oncogenic	miR-217	KRAS	Positive regulation of tumor growth and survival	[115]
AFAP1-AS1	Oncogenic	miR-384	ACVR1	Induces pancreatic cancer stem cell maintenance	[116]
LINC00511	Oncogenic	miR-29b-3p	VEGFA	Modulates angiogenesis	[117]
DLEU2L	Tumor suppressor	miR-210-3p	BRCA2	Alleviates chemotherapy resistance of gemcitabine via inactivation of the AKT/mTOR signaling pathway	[118]
LINC01111	Tumor suppressor	miR-3924	DUSP1	Suppresses tumorigenesis, growth, and metastasis via inactivation of the SAPK/JNK signaling pathway	[125]
GAS5	Tumor suppressor	miR-221	SOCS3	Suppresses cell proliferation, invasion, migration, and gemcitabine resistance by inhibiting EMT and the JAK2/STAT3 signaling	[127]

In conclusion, the above academic results suggest that identified lncRNAs can be beneficial to diagnosis as well as prognosis towards pancreatic cancer, while the lncRNA-mediated ceRNA regulatory network, namely lncRNA/miRNA/mRNA axis, is expected to become a potential therapeutic target for pancreatic cancer.

## Data Availability

The data used to support the findings of this study are available from the corresponding author upon request.
